# Has risk management plan system influenced the speed of package insert revisions in Japan?

**DOI:** 10.3389/fmed.2024.1465313

**Published:** 2024-10-29

**Authors:** Natsuko Kameyama, Aoi Hosaka, Hideki Maeda

**Affiliations:** ^1^Department of Regulatory Science, Graduate School of Pharmaceutical Science, Meiji Pharmaceutical University, Tokyo, Japan; ^2^CMIC Co., Ltd., Tokyo, Japan; ^3^Department of Regulatory Science, Faculty of Pharmacy, Meiji Pharmaceutical University, Tokyo, Japan

**Keywords:** risk management plan in Japan, risk management plan, package insert revision, adverse reaction, drug safety, pharmacovigilance

## Abstract

**Introduction:**

The system of Risk Management Plan in Japan (J-RMP) is a relatively new system, implemented in 2013; thus, its effect on safety measures is still unclear. One of the purposes of J-RMP is to enhance the postmarketing safety measures to be ensured by publishing J-RMP and sharing information on risk management among healthcare professionals. We hypothesized that this might enable information about postmarketing adverse events to be accumulated rapidly, potentially accelerating the identification of adverse reactions (ARs). Herein, we focused on the speed of adding clinically significant ARs (CSARs) to package inserts (PIs) as an indicator of the rapidity of AR identification, investigated the impact of the J-RMP system on PI revisions.

**Methods:**

We investigated the “Notice of Revision of Precautions” on the website of Pharmaceuticals and Medical Devices Agency (PMDA), targeting PI revisions with the addition of CSARs from April 2003 to March 2023, which corresponds to 10 years before and after J-RMP implementation in April 2013. We created an original database from public information of PMDA and investigated the speed of adding CSARs to PIs.

**Results:**

Comparing the time lapse from drug approvals to PI revisions after J-RMP implementation (149 cases) to that before implementation (318 cases), the median value was 32 months for both. Regarding the time lapse when the additional CSARs were listed and unlisted as safety concerns at the time of approvals, it was 35 months vs. 32 months (14 cases vs. 126 cases, *p* = 0.7820), with no statistically significant difference. Conversely, there were significant differences within each AR and each drug therapeutic category.

**Discussion and conclusions:**

This study revealed that the rapidity of risk identification as ARs was not affected by J-RMP, and it may be affected by the characteristics of each AR and each drug therapeutic category. It is expected that other J-RMP benefits, such as risk prevention before the occurrence, will be utilized to further develop strategies for the effective utilization of the J-RMP for safety measures in Japan.

## 1 Introduction

The Risk Management Plan (RMP) in Japan is a document that indicates the risk management of drugs from the development phase to the postmarketing phase. It comprises the following three elements for individual drugs: safety concern, pharmacovigilance activities, and risk minimization activities (1). “Risk Management Plan Guidance,” which was issued in 2012, is applicable to new drugs for which approval applications were submitted on or after April 1, 2013, and requires the creation of RMP in Japan (2). The effect of the J-RMP system (which is a relatively new system) on safety measures is still unclear. Given that it has been more than 10 years since J-RMP was implemented in 2013, we believe it is meaningful to investigate the impact of J-RMP on safety measures and evaluate its effectiveness. One of the purposes of J-RMP is to enhance the postmarketing safety measures to be ensured by publishing J-RMP and sharing information on risk management among healthcare professionals, leading to understand activities as risk management (3). Consequently, it potentially increases spontaneous reports as medical professionals understand the significance of adverse event reporting or recognize it as a risk or insufficient information. Thus, we hypothesized that this might enable information about postmarketing adverse events to be accumulated faster, potentially accelerating the identification of adverse reactions (ARs). However, to the best of our knowledge, no study has been conducted to determine the impact of J-RMP on the rapidity of identifying ARs. In this study, we focused on the speed of adding clinically significant ARs (CSARs) to package inserts (PIs) as an indicator of the rapidity of AR identification and investigated the impact of the J-RMP system on PI revisions.

## 2 Materials and methods

In this study, we investigated the “Summary of Investigation Results” attached to the “Notice of Revision of Precautions” on the website of Pharmaceuticals and Medical Devices Agency (PMDA) (4). “Notice of Revision of Precautions” is a list of notification based on which manufacturers revise their PIs. We targeted PI revisions with the addition of CSARs from April 2003 to March 2023, which corresponds to the 10 years before and after J-RMP implementation in April 2013. The PI revisions from April 2013 to March 2023 were included as PI revisions after RMP implementation, and PI revisions of drugs with no RMP at the time of approval were excluded from the analysis. The PI revisions from April 2003 to March 2013 were included as PI revisions before RMP implementation. We created an original database and first checked the background characteristics of PI revisions for additional CSARs and therapeutic category of drugs. Next, we compared the speed of adding CSARs to PIs with respect to (1) before and after RMP implementation, and (2) listed and unlisted CSARs as the safety concerns at the time of approval. We also investigated the speed by each CSAR and each therapeutic category of drugs.

The speed of adding the CSAR to the PI was defined as the time from the initial approval for new active ingredients of the drug (s) to the date of issuance of the “Notice of Revision of Precautions.” When comparing such speed of PI revisions, “after RMP implementation” refers to PI revisions from April 2013 to March 2023 for products first approved after April 2013, with RMPs at the time of approval, and “before RMP implementation” refers to PI revisions from April 2003 to March 2013 for products first approved after April 2003, with no RMPs. If “Draft drug risk management plan (5)” was included in the review report at the time of approval, it was determined that the RMP was created at the time of approval. Whether or not the CSARs were listed as the safety concerns was also checked by “Draft drug risk management plan.”

This study was conducted per the Strengthening the Reporting of Observational Studies in Epidemiology (STROBE) reporting guidelines (6) for cross-sectional studies. All statistical analyses were performed using the analytical tools of JMP Pro 15, with two-sided *p*-values less than 0.05 being considered statistically significant. The Wilcoxon rank sum test was used to perform comparisons between quantitative data while the Chi-square test was used to perform comparisons between categorical data. CSARs were coded using MedDRA (7) ver. 26.0 and classified by System Organ Class. Therapeutic drug categories were classified according to the Japanese Standard Classification of Products (8).

## 3 Results

The most common CSARs after RMP implementation were “Infections and infestations,” “Skin and subcutaneous tissue disorders,” and “Hepatobiliary disorders,” classified by System Organ Class with MedDRA (7). As for CSARs before RMP implementation, “Hepatobiliary disorders,” “Nervous system disorders,” and “Skin and subcutaneous tissue disorders” were the most common. Regarding the therapeutic category of the drugs, “Other oncology drugs,” “Antidiabetic drugs,” and “Metabolic drugs not elsewhere classified,” were the most common after RMP implementation, and “Other oncology drugs,” “Metabolic drugs not classified elsewhere” and “Antiviral drugs” were the most common before RMP implementation. Table 1 shows the background characteristics of the PI revisions for the addition of CSARs.

**Table 1:** Background characteristics of package insert revisions for adding clinically significant adverse reactions.

		After RMP implementation (1)	Before RMP implementation (2)
Additional adverse reactions	Total (*N*)	149	318
Additional adverse reactions (SOC)	Infections and infestations	19	14
	Skin and subcutaneous tissue disorders	18	32
	Hepatobiliary disorders	17	33
	Immune system disorders	14	22
	Metabolism and nutrition disorders	14	15
	Gastrointestinal disorders	10	25
	Blood and lymphatic system disorders	9	29
	Respiratory, thoracic and mediastinal disorders	8	29
	Nervous system disorders	3	33
	Others	39	99
Therapeutic category of drugs	Other oncology drugs	66	72
	Antidiabetic drugs	28	16
	Metabolic drugs not classified elsewhere	15	31
	Antiviral drugs	12	27
	Synthetic antibacterial drugs	0	20
	Psychoneurotic drugs	1	18
	Vaccines	2	17
	Others	25	120

(1) Package insert revision for 10 years (from April 2013 to March 2023) for products first approved after April 2013 and for which the RMP was published at the time of approval. (2) Package insert revision for 10 years (from April 2003 to March 2013) for products first approved after April 2003.

Comparing the PI revisions after RMP implementation with before implementation, the number of CSARs added to the revised PIs was 149 vs. 318, and the median time from approvals to PI revisions was 32 months for both. Additionally, for 140 of the 149 cases after RMP implementation, excluding nine cases having no information on safety concerns at the time of approvals, we investigated the speed of PI revisions. Comparing when the additional CSARs were listed and unlisted as safety concerns at the time of approvals, the number of CSARs was 14 vs. 126, and the median time from approvals to PI revisions was 35 months vs. 32 months (*p* = 0.7820), and these variables did not differ significantly from each other. A comparison of the speed of adding CSAR to the PI is shown in Table 2.

**Table 2:** Comparisons of the time lapse from drug approval to the addition of adverse reactions in package insert revisions.

Addition of adverse reactions		*N*	Median time (month)
After RMP implementation (1)	Total	149	32
	Listed as safety concerns at the time of approval	14	35
	Unlisted as safety concerns at the time of approval	126	32
	No information	9	
Before RMP implementation (2)	Total	318	32

(1) Package insert revision for 10 years (from April 2013 to March 2023) for products first approved after April 2013 and for which the RMP was published at the time of approval. (2) Package insert revision for 10 years (from April 2003 to March 2013) for products first approved after April 2003.

Conversely, median interval from approvals to PI revisions for each CSAR was significantly shorter for “Metabolism and nutrition disorders” (14 cases, 10 months, *p* < 0.0001), and longer for “Blood and lymphatic system disorders” (9 cases, 55 months, *p* = 0.0413) and “Eye disorders” (4 cases, 79.5 months, *p* = 0.0234; Table 3).

**Table 3:** Comparisons of the time lapse from drug approval to the addition of adverse reactions in package insert revisions (by each adverse reaction).

		After RMP implementation	Median time (month)	*p*-value
Additional adverse reactions	Total (*N*)	149	32	
Additional adverse reactions (SOC)	Infections and infestations	19	48	0.4222
	Skin and subcutaneous tissue disorders	18	36	0.4238
	Hepatobiliary disorders	17	53	0.1278
	Immune system disorders	14	29	0.6534
	Metabolism and nutrition disorders	14	10	<0.0001
	Gastrointestinal disorders	10	39	0.4168
	Blood and lymphatic system disorders	9	55	0.0413
	Respiratory, thoracic and mediastinal disorders	8	38.5	0.8234
	Cardiac disorders	8	18	0.2485
	Musculoskeletal and connective tissue disorders	6	23.5	0.1672
	Vascular disorders	5	26	0.7279
	Investigations	5	12	0.2966
	Endocrine disorders	4	48	0.1535
	Eye disorders	4	79.5	0.0234
	Nervous system disorders	3	15	0.13
	Psychiatric disorders	3	25	0.437

The median interval from approval to PI revision by each drug category was significantly shorter for “Antidiabetic drugs” (28 cases, 17 months, *p* = 0.0106) and “Antiviral drugs” (12 cases, 15 months, *p* = 0.0088), and significantly longer for “Metabolic drugs not classified elsewhere” (15 cases, 59 months, *p* = 0.0143) and “Drugs for digestive ulcers” (8 cases, 58 months, *p* = 0.0087; Table 4).

**Table 4:** Comparisons of the time lapse from drug approval to the addition of adverse reactions in package insert revisions (by each therapeutic category of drugs).

		After RMP implementation	Median time (month)	*p*-value
Additional adverse reactions	Total (*N*)	149	32	
Therapeutic category of drugs	Other oncology drugs	66	33	0.3887
	Antidiabetic drugs	28	17	0.0106
	Metabolic drugs not classified elsewhere	15	59	0.0143
	Antiviral drugs	12	15	0.0088
	Drugs for digestive ulcers	8	58	0.0087
	Other hormone drugs	7	46	0.4116
	Other central nervous system drugs	3	29	0.5565

## 4 Discussion

The speed of PI revisions is instrumental in the prompt identification of risks as ARs to improve awareness and patient safety. In this study, we investigated the impact of the J-RMP system on the revisions of PIs, focusing on PI revision speed. This is because we expected that if risks were appropriately managed using J-RMPs, they could be identified as ARs more rapidly, and PI revisions could be faster. We assumed that J-RMP potentially increase spontaneous reports as healthcare professionals understand the importance of adverse event reporting or recognize it as a risk or insufficient information, leading to fast PI revision speed. However, the results revealed that the implementation of the J-RMP system or description as safety concerns at the time of approvals did not affect the PI revision speed regarding the addition of CSARs. As a side note, in the study examining the relationship between the revision of the information in the CSARs section in PI and the description in J-RMP at the time of drug approval, the median time from drug approval to PI revisions was 29.5 months (9), which was nearly the same as that in our study (32 months). One of the reasons for J-RMP not affecting the rapidity of risk identification as ARs in our study is potentially because healthcare professionals take precautions for reducing the risk, making ARs less likely to occur, and slowing down PI revision speed. Concerning the hypotheses of this study, we focused on the possible publication of the J-RMP that might increase the speed of collecting ARs and PI revision speed; however, risk prevention measures can slow down the PI revision speed, thereby affecting the results. Future studies on the impact of risk minimization measures on PI revision speed will be of interest.

Although the J-RMP did not affect the rapidity of risk identification for ARs, it is known to have other advantages. A study by Saito et al. revealed that there is a strong relationship between ARs listed as safety concerns at the time of approval and those being added to the PIs as CSARs postapproval, indicating that safety concerns could potentially induce severe ARs. This suggests that safety concerns in J-RMPs constitute important drug information, and it is expected that medical professionals will contribute to the prevention of severe ARs in patients by utilizing J-RMPs in addition to PIs (10). Furthermore, “Risk minimization activities” of the J-RMP are also important elements for healthcare professionals because they describe measures to minimize the patient’s risk (11). One of the purposes of the J-RMP is to prevent risks before they occur; however, the low usage rate of the J-RMP in clinical settings has been an issue (12). However, in Japan, the medical fee regulations were recently revised in 2024 to include a provision that medical fee points will be increased if sufficient safety instructions are provided using RMP materials at the time of dispensing (13). According to precedents, regulatory renovation had an obvious effect on Pharmacovigilance Planning (PVP). For example, the publication of the revised Good Post-marketing Study Practice in 2017 (14, 15) and the procedure for developing Postmarketing Surveillance plans in 2018 (16) had a clear impact on PVP shown in J-RMP; the proportion of drugs with efficacy issues decreased, safety issues with additional activity also decreased, and database studies increased in contrast (17). Therefore, it is expected that the revision of medical fee regulation will also promote the use of the J-RMP in clinical settings to mitigate risks. Moreover, J-RMP consolidates risk management into one document to ensure that risk assessments are performed (3), which purpose is different from the RMP in the EU (EU-RMP) (18–21) or risk evaluation and mitigation strategies (REMS) in the US (22), as the EU-RMP lists only safety concerns that require particular attention and REMS are mandatory for only some products. One advantage of the J-RMP is that it allows both regulatory authorities and pharmaceutical companies to conduct risk assessments easily and reliably with one document. However, it has been more than a decade since the implementation of the J-RMP, and there are some preparations for which the J-RMP has been terminated at re-examination. Therefore, a future challenge will be how to implement risk management after J-RMP termination (23).

Conversely, there were significant differences in the PI revision speed by each AR and drug category, suggesting that the speed of AR identification may be influenced by the characteristics of each AR and drug effect. The PI is revised based on AR accumulation in Japan/overseas, revisions of CCDS/overseas labeling, and information on overseas measures (24). Of these, AR accumulation in Japan is recorded in terms of the “number of domestic cases,” the “number of cases in which a causal relationship cannot be ruled out,” and the “number of fatal cases” over the last 3 years. It is possible that the accumulation speed of cases and the ease of causality assessment may influence the PI revision speed (25). In “Metabolism and nutrition disorders,” where the PI revision was faster, 12 out of the 14 CSARs were ketoacidosis and dehydration associated with antidiabetic drugs (SGLT2 inhibitors). Moreover, in “Antidiabetic drugs” with faster PI revision, 25 out of the 28 CSARs were Fournier’s gangrene, ketoacidosis, sepsis, and dehydration associated with SGLT2 inhibitors. The common denominator here is that the patients are many (26) and that causality can be easily assessed based on the drug’s mechanism of action (27, 28), which is likely why the PIs were revised quickly. As for “Antiviral drugs” with faster PI revision, four out of 12 CSARs were associated with drugs for influenza A or B virus infection, and one CSAR was associated with drugs for Herpes zoster infection, and the patients were numerous (29, 30). Regarding the two CSARs of anaphylaxis associated with drugs for SARS-CoV-2 infectious diseases, it may be easier to assess the causal relationship because anaphylaxis occurs immediately after exposure to the causative substances (31). As for CSARs with slower PI revision, seven out of nine CSARs of “Blood and lymphatic system disorders” were associated with molecular-targeted anticancer drugs, five of those were associated with immune checkpoint inhibitors, and three out of four CSARs of “Eye disorder” were uveitis associated with immune checkpoint inhibitors (32). The reason for the slower PI revision could be the relatively new mechanism of action of these drugs, which makes it difficult to assess AR causality with these drugs, and there were not many patients receiving the drugs. However, there were no clear features of other ARs or drug categories. In addition, it is possible that PI revisions regarding similar ARs associated with similar drug classes were coincidentally performed simultaneously. Further investigations using larger samples are necessary.

Nevertheless, our study has some limitations. This study targeted PI revisions with the addition of CSARs, which are clinically important and are frequently revised for analysis, and did not include the revision of other sections such as “Other adverse reactions” as well as “Precautions” or “Warnings,” etc. In addition, of the CSAR section revisions, only the new AR terms was counted, and the revision of frequency, intensity such as severity, or outcomes such as death was not counted, because the different wordings of these elements could have obscured the visual judgment to include them or not; the results may have been affected if these had been included to the analysis. In addition, regarding the comparison between findings before and after J-RMP implementation, there may be differences in the drug safety system or the procedure for PI revisions between the target periods, which may have affected the speed of the PI revision process. To minimize this effect, we also compared the PI revision speed only after J-RMP implementation, between when additional CSARs had been included as safety concerns at the time of drug approvals and when they had not been included. Finally, the COVID-19 pandemic potentially affected the results of this study. The number of PI revisions with the addition of CSARs was 40 in 2019 (prepandemic) and 13 in 2020 (postpandemic). However, considering that the number was around 10 in other years, with not much change, the difference in numbers before and after the pandemic could be a coincidence.

## 5 Conclusion

In conclusion, the implementation of J-RMP and safety concerns did not affect PI revision and its speed. However, the J-RMP has other benefits, such as the prevention of risks before they occur and the reliability of risk assessment in one document. These benefits are expected to be utilized to further develop strategies for the effective utilization of the J-RMP for safety measures in Japan.

## Data Availability

The raw data supporting the conclusions of this article will be made available by the authors, without undue reservation.
